# Bacterial Nanocellulose Effect into Wettability and Thermal Stability of Carbon Fiber via Layer-by-Layer for LED Circuit Application

**DOI:** 10.1021/acsabm.5c02197

**Published:** 2026-01-12

**Authors:** Maurelio Cabo, Nitin More, Kyle Nowlin, Ram Mohan, Dennis LaJeunesse

**Affiliations:** † Department of Nanoscience, Joint School of Nanoscience and Nanoengineering, University of North Carolina Greensboro, Greensboro, North Carolina 27401, United States; ‡ Department of Nanoengineering, Joint School of Nanoscience and Nanoengineering, 3616 North Carolina Agricultural and Technical State University, Greensboro, North Carolina 27401, United States; § Department of Mechanical Engineering, 3616 North Carolina Agricultural and Technical State University, Greensboro, North Carolina 27411, United States

**Keywords:** Bacterial Nanocellulose, Hydrophobic, Carbon Fiber, Hybrid Composite, LED, Thermal Stability

## Abstract

This study aimed to investigate how bacterial nanocellulose (BNC) affects the wettability and thermal stability in carbon-fiber (CF) polymer composites. CF/BNC laminates were fabricated through layer-by-layer hot pressing and evaluated by using contact-angle measurements, thermogravimetric analysis, and low-voltage electrical testing. The CF/BNC interface enhanced nanoscale interlocking, increasing the contact angle from 79.6° to 106.46°. Thermal stability improved, as shown by final degradation temperature and higher residual mass, and electrical tests using a light-emitting diode (LED) confirmed efficient current flow with minimal voltage drop. The results demonstrate a pathway for multifunctional, hydrophobic, and thermally stable composites for low-voltage electronic applications.

## Graphical abstract

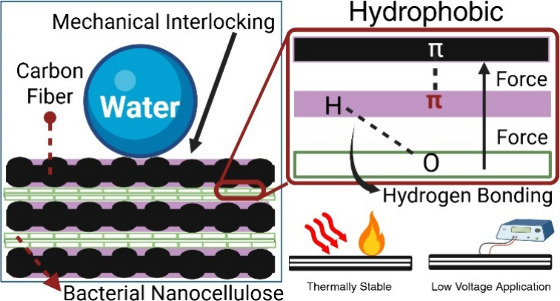

Carbon fiber reinforced polymers (CFRPs) are modern materials with high modulus and strength, ideal for applications requiring high strength and stiffness at a low weight. Typically used in aerospace, advanced engineering projects, cars, trains, sports equipment, ships, and wind energy over the past five decades^1.^ However, despite their impressive structural properties, carbon fibers cured with thermoset resin still open for more improvement, particularly in their surface chemistry^2,3^ and thermal stability in oxygen-rich atmospheres,^4^ which can outperform its current capacities in harsh or multifunctional environments.

One of the persistent challenges with carbon fibers cured with epoxy resin is the relatively inert and hydrophilic surface, which leads to poor interfacial adhesion and weak resistance to moisture. In recent years, researchers have explored surface modification techniques, including plasma treatment,^5^ oxidation,^6^ and the use of functional nanofillers,^7^ to address these limitations. Surface modification not only enhances fiber–matrix adhesion but also opens pathways for improving wettability, thermal conductivity, and chemical resistance.

Though carbon fiber is highly thermally stable in an oxygen-free environment, its composites when combined with conventional thermosetting and thermoplastic resins, such as epoxy and polypropylene, and when exposed in an oxygen-rich environment, may offer initial strength but often lack the thermal endurance required for long-term structural performance. Recent advancements have focused on hybrid reinforcement strategies—combining carbon fiber with cellulose that provides a synergistic effect in improving heat resistance and delaying decomposition onset.^8^ Beyond technical performance, as plastic pollution and nonbiodegradable waste continue to pose severe ecological threats, there is growing pressure to develop composites that are not only high-performing but also environmentally responsible. Commercial carbon fiber composites rely on thermoplastic matrices that are difficult to recycle and contribute to long-term plastic pollution.^1^ That is why cellulose came into the picture because replacing these polymers with bioderived, biodegradable, or recyclable alternatives has become a crucial research direction. Several recent studies incorporating cellulose focused more on bridging the gap between mechanical reinforcement and eco-compatibility in composite systems^9^ but still leave room how biopolymer improves wettability and thermal stability without complex surface modifications in which our study has contributed. Herein, when we integrated into carbon fiber composites, BNC has the potential to enhance interfacial bonding, improve thermal stability, and control the electrical performance without further surface modification.

In this context, bacterial nanocellulose (BNC) emerges as a promising green alternative.^10^ BNC is a highly pure, mechanically strong, and biodegradable nanomaterial synthesized by microbial fermentation.^11^ Thus, developing CFRCs with BNC is both a technological necessity and an ecological imperative. This study aims to explore a new CF/BNC hybrid composite providing how bacterial nanocellulose (BNC) affects interfacial synergy with carbon fibers and establishes a framework for designing multifunctional, thermally stable, and hydrophobic polymer composites for next-generation low-voltage electronic applications.

Figure S1 shows that the composite samples were fabricated through a layer-by-layer approach, incorporating carbon fiber (CF) sheets and bacterial nanocellulose (BNC) while the Table S1 discloses the sample composition and curing parameters used. The three samples prepared include a single-layer carbon fiber sample (1CF), a hybrid structure with three layers—two CF layers and one BNC layer (2CF/1BNC)—and a more complex composite with five layers—three CF layers and two interleaved BNC layers (3CF/2BNC). Each sample was impregnated and cured with epoxy resin to form a consolidated composite structure. Despite the introduction of BNC layers, the flexibility of carbon fiber composites remained largely unaffected, Figure 1. Visual inspection of the bent samples, Figure 1(A.1, B.1, C.1), shows that all three samples show an apparent flexibility even when the BNC was introduced.

**Figure 1:**
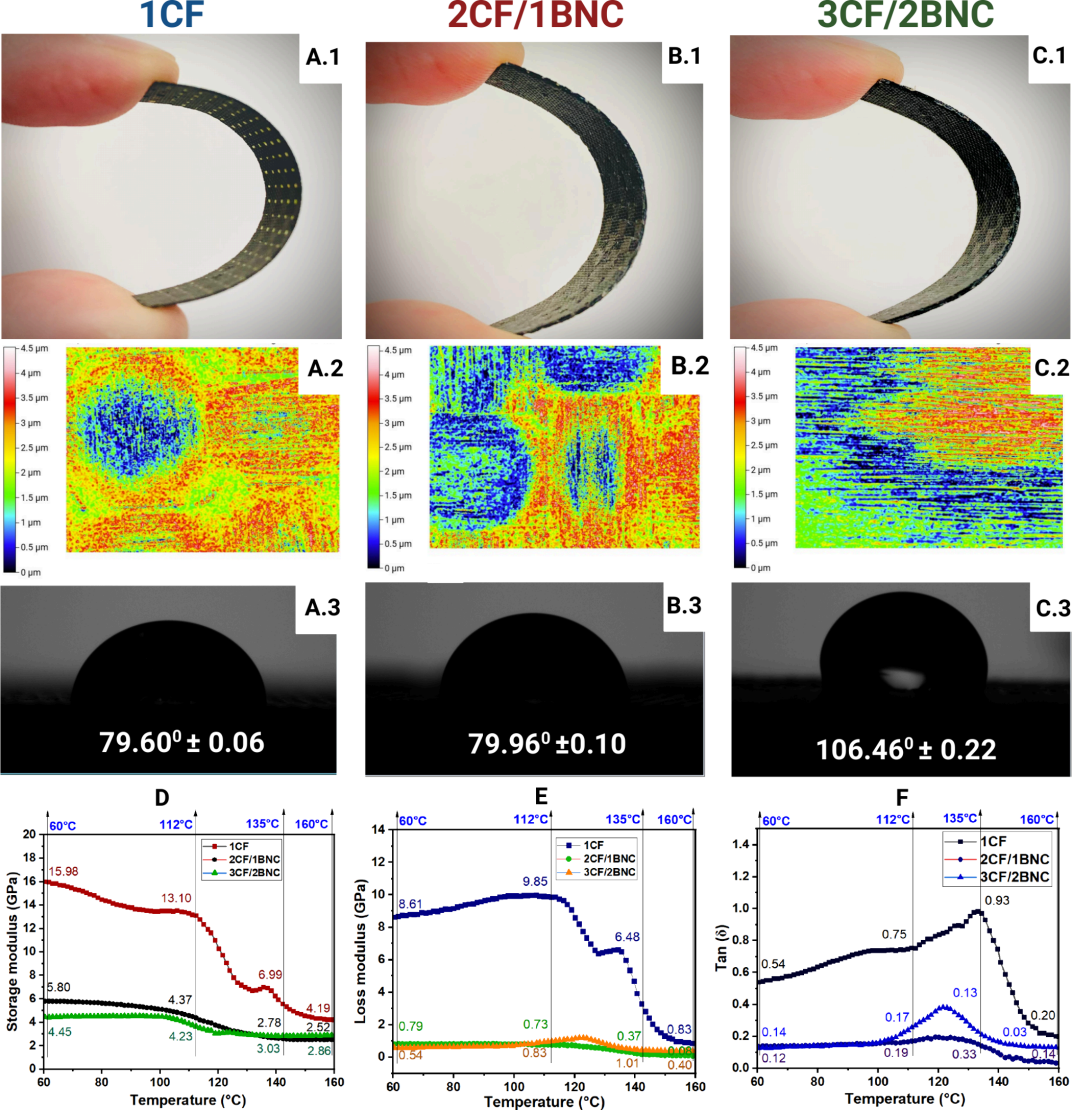
Samples prepared as the following: one layer of carbon fiber, 1CF (A.1); two layers of carbon fibers with 1 layer of bacterial nanocellulose, 2CF/1BNC (B.1); and three layers of carbon fibers with two layers of bacterial nanocellulose, 3CF/2BNC (C.1). Curing process and layer by layer fabrication changed the surface morphology of the samples as shown by KLA-Zeta Analyzer (A.2, B.2, C.2). Measured contact angle for each sample in triplicate reported with SD (A.3, B.3, C.3). DMA analysis measuring the following: storage modulus, *G*′ (D); loss modulus, *G*″ (E), and tan δ (F) in the following temperature ramps: 60, 112, 135, and 160 °C for frequency of 1 Hz. Sample dimensions were disclosed in Table S1.

To better evaluate surface morphology, heat maps using KLA-Zeta analysis (Figure 1(A.2, B.2, C.2)) were generated. These reveal differences in surface roughness across the samples, suggesting that the curing process and the number of layers influence topographical outcomes. The increase in layers, particularly with BNC, appears to elevate the carbon fibers surface, from 1.261 up to 2.061 μm and depth from 0.94 to 1.574 μm as disclosed in Table S2. The heatmap also shows the disappearance of a more pronounced pattern which means the distance between each fiber was totally covered by succeeding layers and resins which indicates by intense blue areas.

In Figure S2­(A), mass, volume, and density measurements show an increasing trend across the samples, consistent with the added layers of carbon fiber and bacterial nanocellulose (BNC). While the increase in volume and density changes is moderate, this led to our hypothesis to observe not only how the surface morphology changes but also how its surface hydrophobicity may improve. Surprisingly, in Figure 1(A.3, B.3, C.3), which displays contact angle measurements, the 1CF and 2CF/1BNC samples exhibit almost similar contact angles, 79.60° and 79.96°, respectively, indicating relatively hydrophilic or marginally wettable surfaces; however, 3CF/2BNC shows a markedly increased contact angle of 106.46°, clearly transitioning the material into the hydrophobic property. This significant increase implies a change in surface structure in spite of no surface modification or pretreatment before layer-by-layer fabrication.

Table S3 discloses the comparison of this result from other known carbon fiber reinforced polymers or composites. This behavior illustrates the interaction mechanisms within the layered composite suggesting hydrogen bonding between BNC and epoxy resin^12^ and π–π stacking between CF and epoxy resin^13^ layers contribute to mechanical interlocking and structural coherence. This layered architecture likely reduces the surface energy and modifies surface roughness—key factors known to enhance water repellency.^14^

Samples evaluation of the mechanical behavior under dynamic and tensile conditions was also characterized. Herein, the Dynamic Mechanical Analysis (DMA) results in Figure 1(D, E, F) reveal how the addition of bacterial nanocellulose (BNC) affects viscoelastic performance across temperatures.

In Figure 1D, the storage modulus (*G*′) represents elastic stiffness—declines for all samples with increasing temperature, as expected. However, the 1CF initially demonstrates the highest modulus, 15.98 GPa at 60 °C, indicating strong interfacial interactions between CF and epoxy resin,^15^ yet the 2CF/1BNC and 3CF/2BNC samples show reduced modulus values, likely due to increased structural damping^16^ from additional BNC layers. Figure 1E, loss modulus (*G*″), and Figure 1F, tan δ, show lower damping behavior for BNC-containing samples, particularly for 3CF/2BNC, where the tan δ maximum peak is only 0.33 compared to 0.93 in 1CF at 135 °C which implies reduced chain mobility due to enhanced interfacial cohesion.^17^ But its value lower than 1.0 confirmed its flexibility form.^18^

In support, the X-ray diffraction (XRD) patterns in Figure S2­(B) show two key crystal phase peaks at 25° (002) and 44° (100). The slight decrease in crystallinity, Table S4, resulted in reduced stiffness and strength of the semicrystalline polymers but enhanced ductility due to fewer secondary intermolecular bonds and greater structural disorder.^19^ Notably, an increase in crystallite size was observed at the 002 planes, from 0.91 in 1CF to 1.56 in 3CF/2BNC, and at the 100 planes, from 1.42 in 1CF to 2.28 in 2CF/1BNC. The functional groups and elemental composition of the samples were determined by Fourier-transform infrared spectroscopy (FTIR), Figure S2­(C) and scanning electron microscopy-energy-dispersive X-ray (SEM-EDX), Figure S3, respectively. The FTIR spectra aligned with previous studies on thermosetting carbon fiber–reinforced plastic (CFRP) using epoxy resin.^20^

Functional groups such as N–H, C=C, N–O, C–N, and C–O–C (oxirane) were attributed to the epoxy resin and curing agent, while O–H, C–H, and C–O were characteristic of carbon fibers and bacterial nanocellulose. The presence of these oxygen-rich groups, while evident in the spectra, supports the suggestion that the incorporation of bacterial nanocellulose ultimately enhanced the hydrophobicity of the composites by limiting the exposure of polar sites and water–surface interactions. While the SEM-EDX confirmed that C, O, and N were the prominent elements. This further revealed that the introduction of bacterial nanocellulose increased the proportion of O. This compositional shift is significant because, although oxygen content increased, much of it became integrated within the crystalline or cross-linked network, reducing the availability of polar groups at surface.^21^ As a result, the material transitioned from being relatively hydrophilic to exhibiting enhanced hydrophobicity, consistent with contact angle results.

For further characterization, we are interested to know if there’s a significant effect on its thermal stability by looking at thermogravimetric analysis (TGA) curves. In Figure 2A, it reveals a significant improvement in residual yield (RY%) for BNC-containing samples. While the 1CF sample retains only 48.98% of its weight at 600 °C, the 2CF/1BNC and 3CF/2BNC composites achieve 63.98% and 72.91% RY, respectively. This improved stability corresponds with the morphology where BNC acts as a reinforcing barrier against thermal degradation which aligns with previous study of how thermally insulating materials based on renewable nanomaterials such as nanocellulose could reduce the energy consumption.^22^

**Figure 2:**
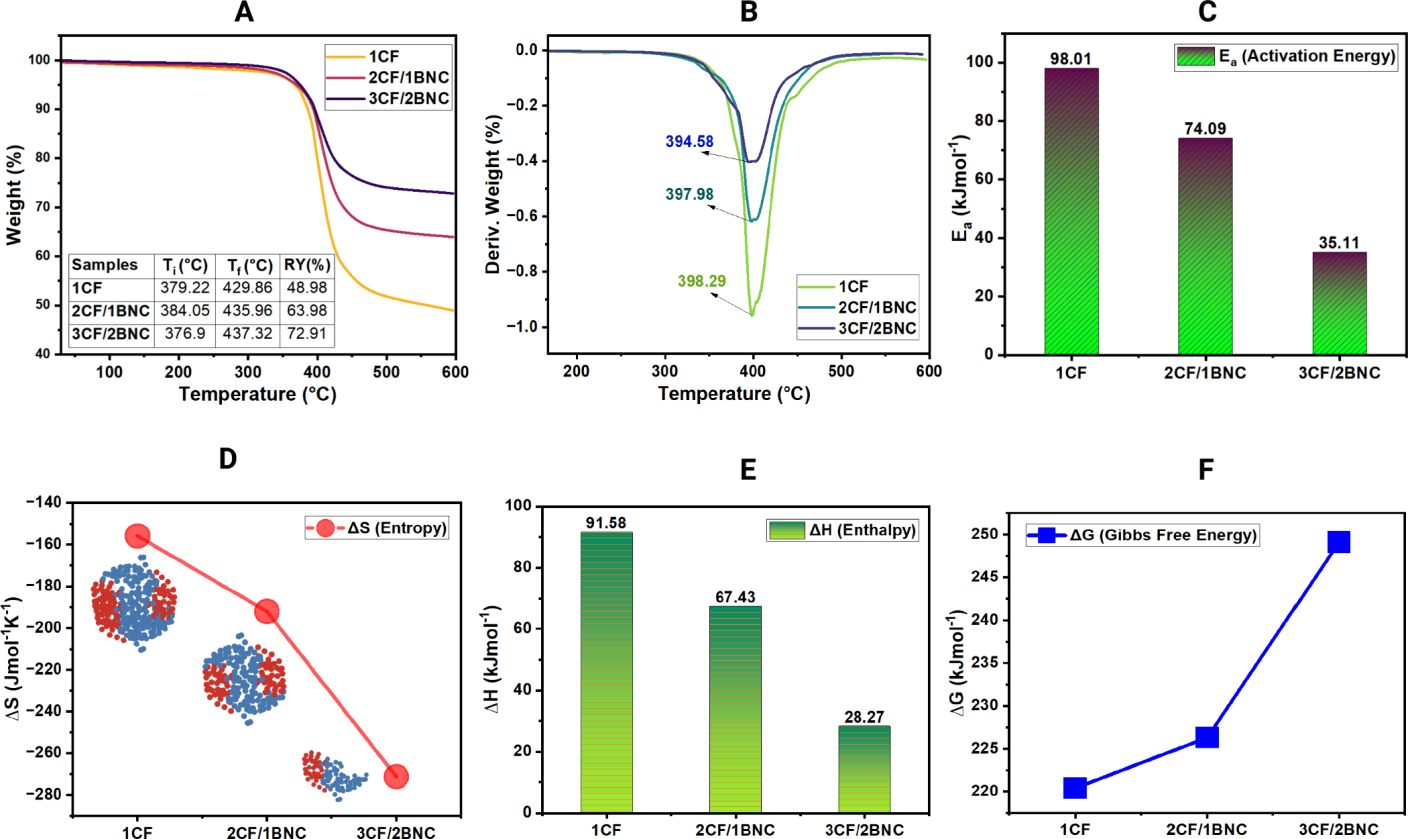
Thermogravimetric curve (A); derivative thermogravimetric curve (B); calculated activation energy (C), entropy (D), enthalpy (E), and Gibbs free energy of the prepared samples (F).

The derivative weight curves, Figure 2B, revealed a slight downward shift in peak degradation temperatures for the BNC-enhanced samples (397.98 °C for 2CF/1BNC and 394.58 °C for 3CF/2BNC) compared to 398.29 °C for 1CF. This deviation from the usual trend is noteworthy, as these same samples demonstrated improved residual yield and a higher end set temperature,^23^ indicating that while the onset of thermal decomposition occurred marginally earlier, the overall thermal stability and char-forming ability were enhanced. Figure 2(C–E) provides kinetic insights, showing a decrease in activation energy (*E*_a_), enthalpy (Δ*H*), and entropy (Δ*S*) with increasing BNC content. The reduction in *E*_a_ (from 98.01 to 35.11 kJ/mol) and Δ*H* (from 91.58 to 28.27 kJ/mol) may initially suggest a less energy-intensive degradation process.

However, this is interpreted as a shift toward a more thermodynamically stable network, where energy barriers are lowered due to improved bonding interactions and uniform resin diffusion.^24^ Interestingly, Figure 2F shows an increase in Gibbs free energy (Δ*G*), indicating greater thermal favorability^25^ for the 3CF/2BNC composite. This thermodynamic behavior suggests that BNC not only stabilizes the composite structurally but also improves its resistance to thermal and environmental stresses.^26^ Table S5 shows the full disclosure of each phase kinetics and thermodynamic parameter result, and Table S6 discloses a comparative study in terms of degradation temperature from the related studies.

In Figure 3 to demonstrate a facile proof-of-concept application, highlighting the electrical conductivity potential of the carbon fiber–bacterial nanocellulose (CF/BNC) layered composites, we integrate these composites into a basic LED circuit powered by a battery source, the samples act as conductive bridges capable of enabling current flow to illuminate a low-voltage LED.^27^

**Figure 3:**
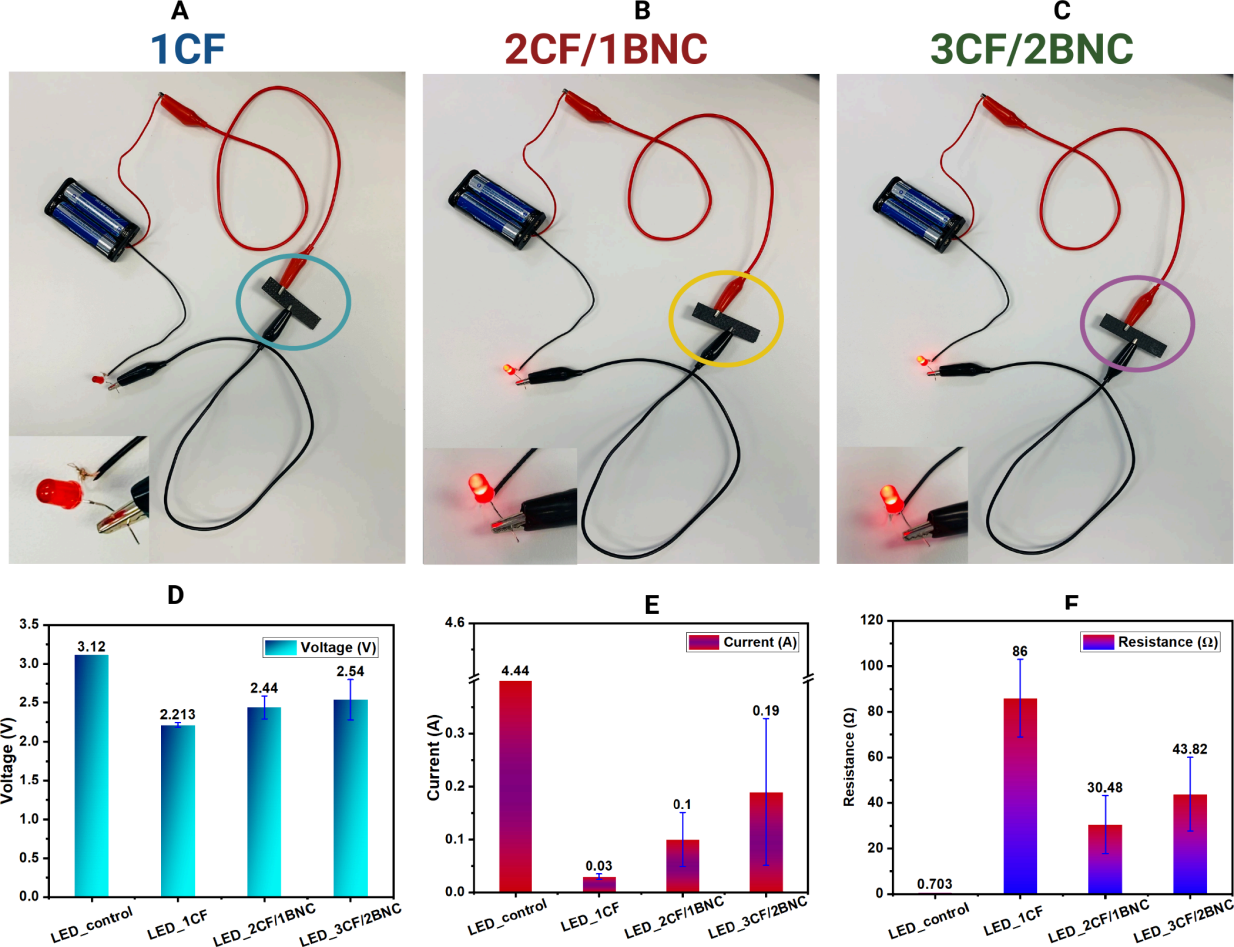
Application for low-voltage LED lights where 1CF (A) did not showing effective electrical conductivity while 2F/1BNC (B) and 3F/2BNC (C) managed to light up the LED light bulbs. Voltage drop (D), controlled current flow (E) and resistance flow (F) supported the target application.

In the visual setup, two out of three composites—2CF/1BNC, Figure 3B, and 3CF/2BNC, Figure 3C—successfully complete the circuit and power the LED, confirming their ability to conduct electricity. LED in 1CF, Figure 3A, failed to have steady light up maybe due to insufficient and unstable current flow.^28^ While the LED brightness varies slightly across samples, this demonstration validates the feasibility of using these composites in simple electronic or sensing applications. Quantitative measurements shown in Figure 3(D–F) further support this functionality. Voltage measurements, Figure 3D, indicate only moderate drops across the composite samples, 2.213 V for 1CF, 2.44 V for 2CF/1BNC, and 2.54 V for 3CF/2BNC, compared to the control voltage of 3.12 V.

These values suggest reasonable voltage retention, despite internal resistance. Current measurements, Figure 3E, reveal that the 3CF/2BNC sample allows the highest current flow, 0.19 A, among the composite samples, followed by 2CF/1BNC, 0.1 A and 1CF, 0.03 A which explains nonsteady LED lighting up. This trend correlates with the observed decrease in resistance, Figure 3F, where the 3CF/2BNC composite shows significantly lower resistance, 43.82 Ω than 1CF, 86 Ω, indicating better electron mobility in the layered architecture.^29^ Additional information was also provided for the calculated resistivity (ρ) and sheet resistance (*R*_s_) as shown in Figure 4.

**Figure 4:**
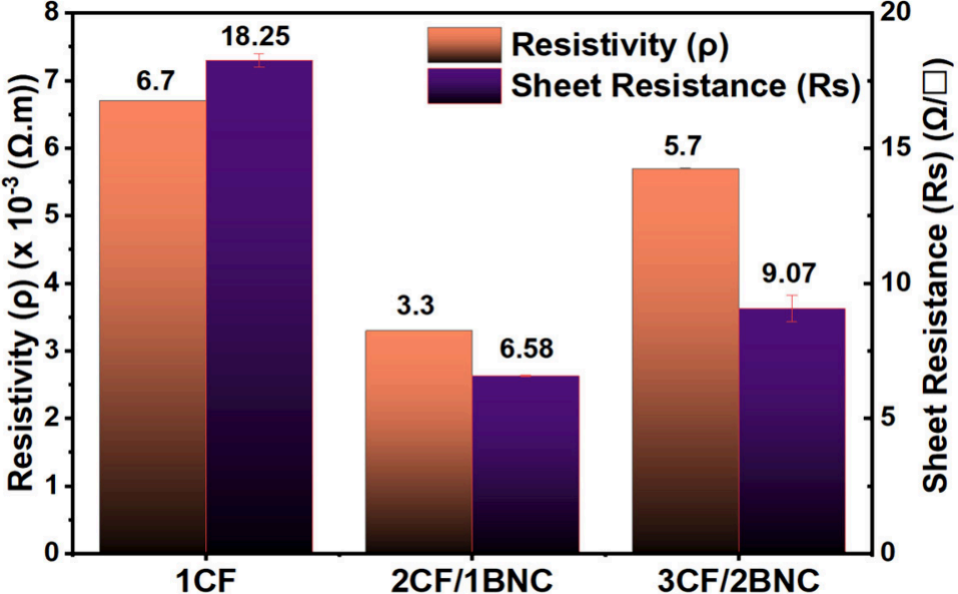
Calculated resistivity (ρ) and sheet resistance (Rs) of the samples.

Although 1CF’s intrinsic resistivity is moderate, its thin structure results in a high effective resistance, preventing enough current from reaching the LED. In contrast, the thicker multilayer samples provide lower sheet resistance and better conductive pathways, allowing sufficient current flow to light the LED. The electrical performance showcased here suggests that the CF/BNC hybrids could serve lightweight, flexible, and low-voltage functional components—such as sensors, conductive coatings, or energy-harvesting layers in wearable or structural bioelectronics using functionalized BNC.^30^

This study presents the development of a carbon fiber–bacterial nanocellulose (CF/BNC) hybrid composite using a simple, scalable layer-by-layer hot-pressing method. Incorporating two layers of BNC between three carbon fiber sheets enhanced surface hydrophobicity and thermal stability without significantly reducing mechanical flexibility as confirmed by storage and loss modulus. A facile electrical test confirmed the composite’s conductivity, with the CF/BNC sample successfully powering a low-voltage LED. The CF/BNC hybrid composite combines thermal and surface functionality and electrical semiconductivity, making it a promising candidate for hybrid composite useful for flexible wearable materials, biosensors, and the next-generation low-voltage electronic applications.

## Supplementary Material



## Notes

Experimental section, culturing, fabrication, and hotpress drying of bacterial nanocellulose and layer by layer structuring of the samples (Figure S1), measured mass, volume and density, enhanced crystallinity shown by XRD, and functional group analysis using FTIR (Figure S2), SEM-EDX images and elemental composition (Figure S3), samples composition and curing parameters used (Table S1), surface topography and roughness measurement (Table S2), comparison of measured contact angle (water) to other existing known carbon fiber reinforced polymer or composites (Table S3), degree of Crystallinity (%) and crystallite size from XRD (Table S4), kinetic and thermodynamic parameters from thermogravimetric analysis (Table S5), comparison of Measured Endset Temperature to other existing known carbon fiber reinforced polymer or composites (Table S6) (PDF)
