# Supplementary material for: Bacterial Nanocellulose Effect into Wettability and Thermal Stability of Carbon Fiber via Layer-by-Layer for LED Circuit Application
Source: ACS Appl Bio Mater. 2026 Jan 12;9(3):1265–71. doi: 10.1021/acsabm.5c02197 (PMC12869473; doi:10.1021/acsabm.5c02197)
Supplement: Supplementary file 1 [file mt5c02197_si_001.pdf]

Supporting Information

Bacterial Nanocellulose Effect into Wettability and Thermal Stability of Carbon Fiber via Layer-by-Layer for LED Circuit Application

[1]Maurelio Cabo Jr., [2]Nitin More, [1,2]Kyle Nowlin, [3]Ram Mohan, *[1]Dennis LaJeunesse

[1]Department of Nanoscience, Joint School of Nanoscience and Nanoengineering, University of North Carolina Greensboro, Greensboro, North Carolina, 27401, USA
[2]Department of Nanoengineering, Joint School of Nanoscience and Nanoengineering, North Carolina Agricultural and Technical State University, Greensboro, North Carolina, 27401, USA
[3]Department of Mechanical Engineering, North Carolina Agricultural and Technical State University, Greensboro, North Carolina, 27411, USA

Corresponding Authors: *drlajeun@uncg.edu

Experimental Section

A. Materials

(D+)Glucose, Yeast extract, NaOH pellets, Na_2HPO_4, Agar powder, and Peptone were purchased from Fisher Scientific (Thermo Fisher Scientific, Waltham, MA, USA). Pellicles of BC were collected from cultures of Gluconacetobacter hansenii (ATCC 23769, American Type Culture Collection, Manassas, VA, USA). Carbon fiber was purchased from Toray Industries, Inc., USA, which is plain weave (T300B-3000-40B), with thickness of 0.24 mm and areal weight of 6 oz (203 g/m^2) and PTFE Teflon Sheet (12”x16”) was purchased from Ubrand, USA. Epon Resin 862 (Viscosity of 25-45 P) and Epicure (Viscosity of 150-300 cP) curing agent were purchased from Sigma Adrich, USA.

B. Fabrication of Bacterial Nanocellulose

Bacterial nanocellulose (BNC) was fabricated in Hestrin-Schramm (HS) media under static culture, followed by NaOH pretreatment and drying. The Gluconacetobacter hansenii strain was cultured in HS media composed of 2% (w/v) D-glucose, 0.5% (w/v) yeast extract, 0.5% (w/v) peptone, 0.27% (w/v) Na_2HPO_4, and 0.125% (w/v) citric acid [1–2]. For inoculation, the strain was first grown on an agar plate (85 mm × 15 mm) containing the same HS composition with 2% agar and incubated at 30 °C for 2 days. A pea-sized colony was then transferred into 500 mL of HS media in a 9 × 13 in. glass dish and incubated at 25 °C for 10 days. The resulting pellicles were treated with 0.1 M NaOH at 95 °C for 1 h to remove bacterial residues and biofilms, rinsed with distilled water to neutral pH (7.0), and stored in deionized water at room temperature. Using Hotpress Model 25-12H, USA, at 35 psi, the wet bacterial nanocellulose with dimension 9 x 13 inches was folded into four and sandwich between the 11x11 cm size cut Teflon sheet [3].

C. Fabrication of CF/BNC Hybrid Composite

Table S1 shows the samples composition and curing parameter used for this study. To maintain the lightweight density of the hybrid composite we only fabricated until 3-ply for CF and 2-ply for BNC. For curing process, per sample, we mix 100:29 ratio of EPON 862 epoxy resin (77.52 wt%/v) to EPICURE curing agent (22.48 wt%/v), using the below equations for a total volume of 10 mL for 1CF, 20 mL for 2CF/1BNC and 40 mL for 3CF/1BNC.

$$wt\%\ of\ EPON\ 862 = \frac{W_{epoxy}}{W_{epoxy} + W_{curing}}\ x\ 100 \quad (1)$$

$$wt\%\ of\ EPICURE\ curing\ agent = \frac{W_{curing}}{W_{epoxy} + W_{curing}}\ x\ 100 \quad (2)$$

A hand lay-up technique was used to distribute the resin and curing agent over each layer, using 10 mL per ply. For each ply, the carbon fiber layer measured 4 × 6 inches, while the dried bacterial nanocellulose layer measured 8 × 8 cm. Density of all of the samples were measured using the classical equation: D = M/V wherein M is the mass of the sample and V is the total volume (L x W x H). A Hot Press (Model 25-12H, USA) operating at 35 psi was used for pre-curing, during and post curing the composite, and a water jet cutter (Model: Flow Mach 300) was used to cut the composite into the desired dimensions for the actual samples and characterization.

D. LED Circuit Set-up

To evaluate the electrical performance of the CF/BNC composites, a simple Light Emitting Dioded (LED) bulb circuit was assembled using a AA battery pack as the power source (1.5V), 1.5V AA Battery Holder Case Box, conductive wires (Alligator Clips,20.5 inches / 22 AWG Copper Wire), and a Latching Rocker Switch ON/Off 2 Pin 2 Position SPST Car Auto Boat Square Rocker Toggle Switch with Pre-Wired AC. Each composite sample was inserted in series as the conductive bridge, replacing the switch, between the battery and a low-voltage LED [4]. Closing the switch completed the circuit, allowing current to pass through the sample and illuminate the LED if conductivity was sufficient. Multimeter was used to measure Voltage, current and resistance. By using the measured resistance, length, width and thickness of samples, we able to compute the resistivity [5] then the calculated resistivity, ρ (Ω·m) was used to determine the sheet resistance [6] using the below equations:

$$\rho = \frac{RA}{L} \quad (3)$$

Where ρ = resistivity; R = resistance; A = cross sectional area of the cut sample; and L = length of the cut sample.

$$R_s = \frac{\rho}{t} \quad (4)$$

Where R_s = sheet resistance; ρ = resistivity; and t = thickness of the cut sample.

E. Characterization Techniques

Contact angles for the samples were performed using a Rame Hart Model 260 Goniometer. Crystallinity analysis was conducted using Rigaku SmartLab equipment with Cu-Kα radiation. A copper source was used at 40 V and 40 mA. The samples were mounted on the sample holder, and the patterns were recorded by running the instrument at a speed of 5°/ min and a 2θ range of 5°–40°. The Scherrer's formula was used to measure the crystallite size:

$$CrystalliteSize = \frac{k\lambda}{W} cos\theta \quad (5)$$

(with a shape factor k = 0.94 was employed to determine the crystallite sizes of samples with full width at half maximum (fwhms, W) and peak centers obtained by fitting the (002) and (100) peaks at the Gaussian function using OriginPro software; here λ is the wavelength of X-ray radiation (0.154 nm) [7]. The lattice spacing (d-spacing) was calculated using Bragg's equation [8-9]:

$$\lambda = 2d_{hkl} * sin\theta \quad (6)$$

The degree of crystallinity was calculated with the help of the following formula:

$$Degree\ of\ Crystallinity = \left(\frac{\text{Area of crystalline peaks}}{Area\ of\ crystalline\ peaks + Amorphous\ peaks}\right) x100 \qquad (7)$$

IR spectra were recorded on a Fourier-transform infrared spectroscopy (FTIR) spectrometer (Agilent 670 FTIR Spectrometer, Santa Clara, CA, USA) under dry air at ambient temperature. The percentage of transmittance spectra was recorded from 4000 to 400 cm^{-1} with 64 scans in each case at a resolution of 4 cm^{-1}.

The scanning electron micrographs were obtained using JEOL JSM-IT800 Schottky FESEM (Zeiss, Jena, Germany). Surface morphologies were assessed while elemental characterization through energy-dispersive X-ray spectroscopy (SEM/EDX) was employed. To do heat map simulation on samples' elevated surface, optical profiler was employed. This benchtop optical profiler is a non-contact, 3D microscope and surface topography measurement system, model: Zeta-20 (KLA Corporation, USA). The thermal stabilities were characterized by using a thermogravimetric analyser (TGA; Perkin Elmer STA 6000, England) within a temperature range of 30 to 600 °C at a rate of 20 °C/min under an air atmosphere. The kinetic parameters were determined using a modified form of the Coats and Redfern model as described [10] in the following equations:

$$ln\,[-\,ln(1-x)] = ln\,(\frac{ART^2}{\beta E_a} - \frac{E_a}{RT}) \qquad (8)$$

where x is the first rate if reaction, A is the pre-exponential factor, β is the heating rate (20 °C/min), R is the general gas constant (8.3143 $Jmol^{-1}\ K^{-1}$), E_a is the activation energy, and T is the temperature (K). Plotting graphs between ln[−ln(1 − x)] vs 1000/T for each phase gave the value of activation energy, and further parameters were determined using basic thermodynamic equations [11].

Entropy change: $$\Delta S = R\left[ln\left(\frac{Ah}{KT}\right)\right] \qquad (9)$$

where h is the Planck constant and K is the Boltzmann constant.

Enthalpy change: $$\Delta H = E_a - RT \qquad (10)$$

Gibbs free energy change: $$\Delta G = \Delta H - T\Delta S \qquad (11)$$

The mechanical-dynamic tests were performed using TA HR20 Rheometer to study the temperature dependence of the viscoelastic properties of these composites in the range from 60 up to 160 °C with frequency of 1 Hz using 25 mm frame size.

F. Statistical Analysis

Contact angle and electrical performance was performed in triplicate, and each response's mean value ± SD was reported. All spectra and graphs were generated using OriginPro 2024b Academic Software.

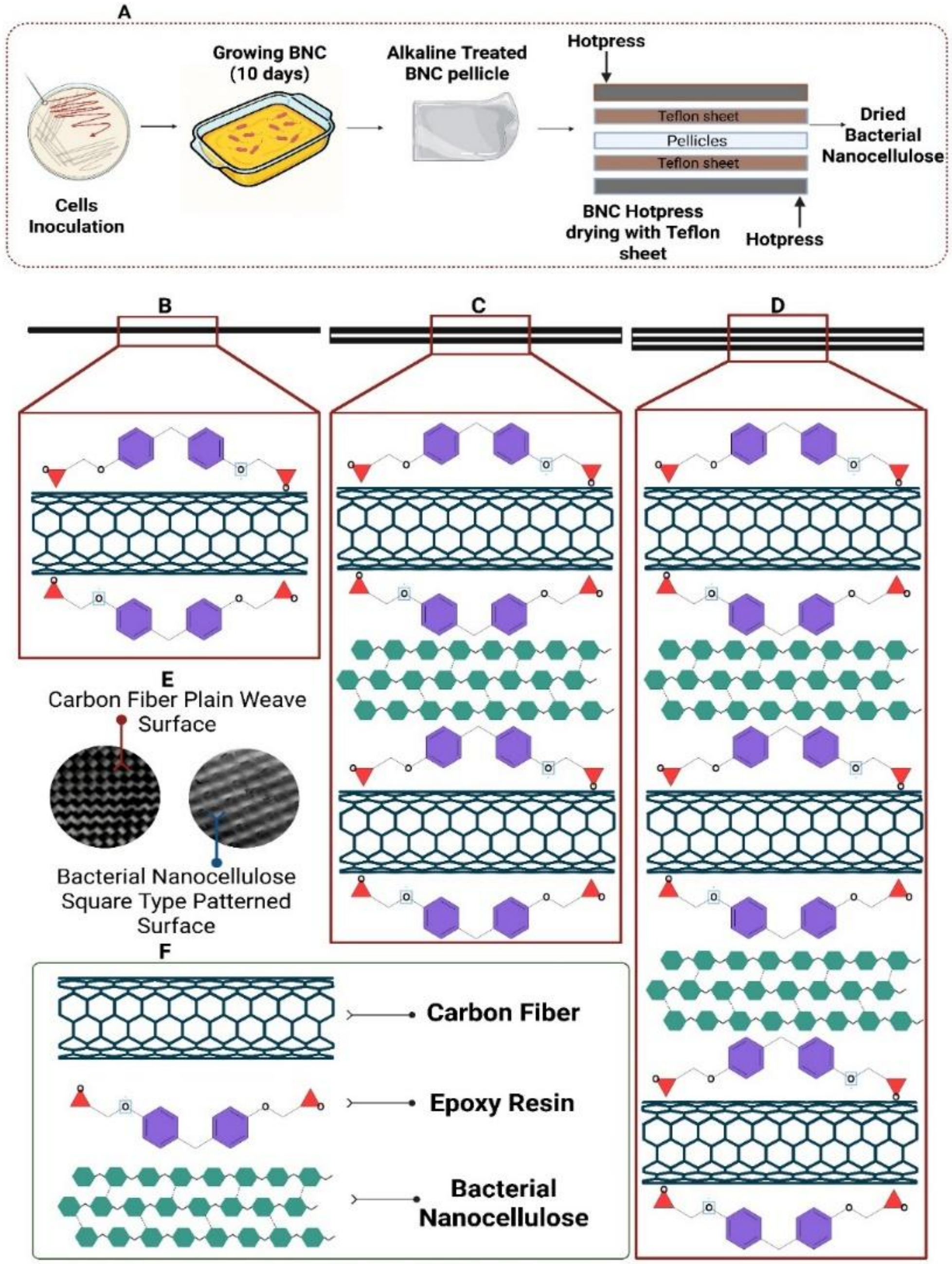

Figure S1: Culturing, fabrication, and hotpress drying of bacterial nanocellulose (A); Layer by layer structuring of the samples: 1CF (B); 2CF/1BNC (C); 3CF/2BNC (D); SEM images of surface morphology carbon fiber and bacterial nanocellulose showing distinct patterns (E); Molecular structure of carbon fiber, epoxy resin, and bacterial nanocellulose (F).

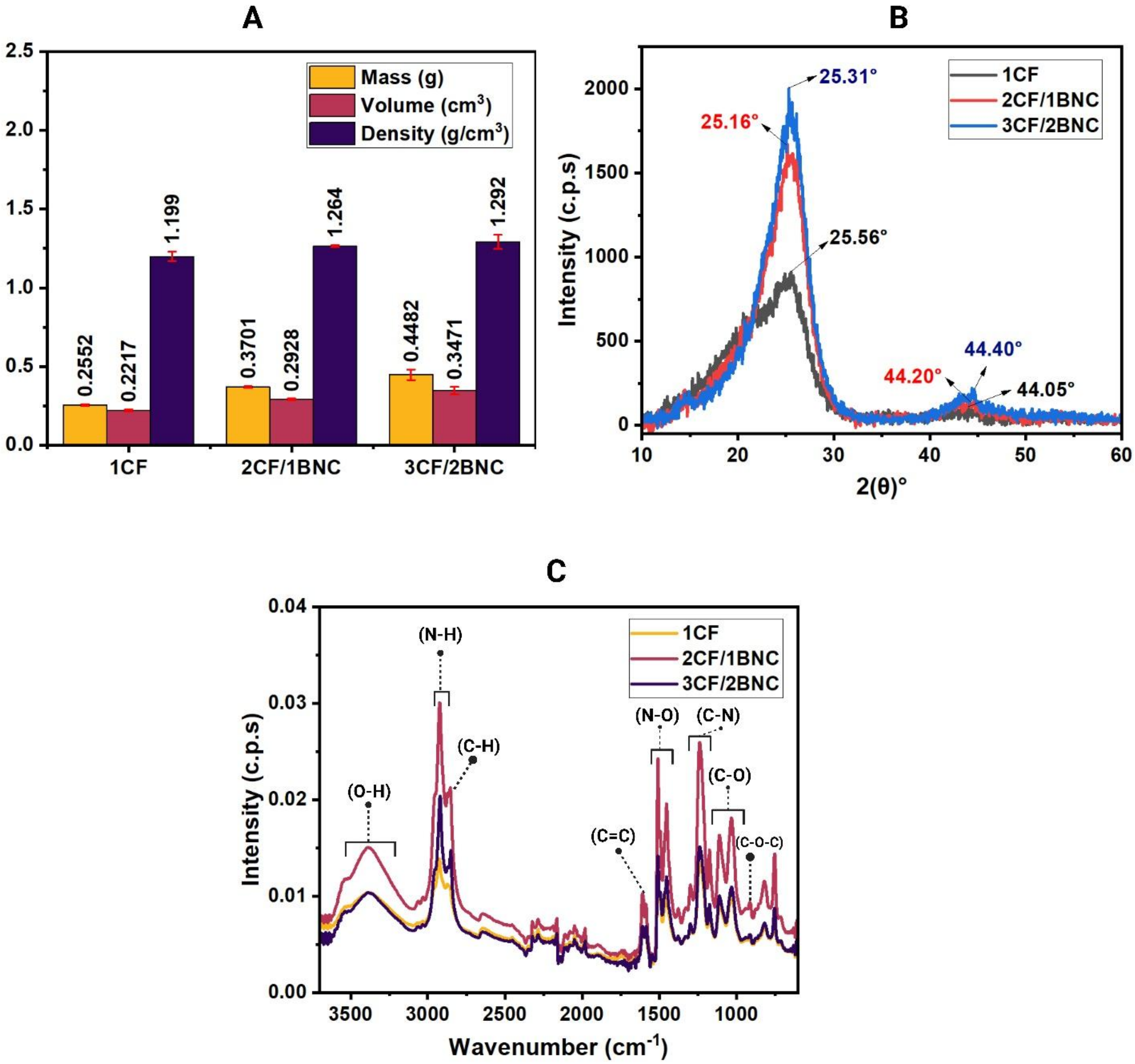

Figure S2: Measured Mass, Volume and Density (A). Enhanced crystallinity showed by XRD (B). Functional group analysis using FTIR (C).

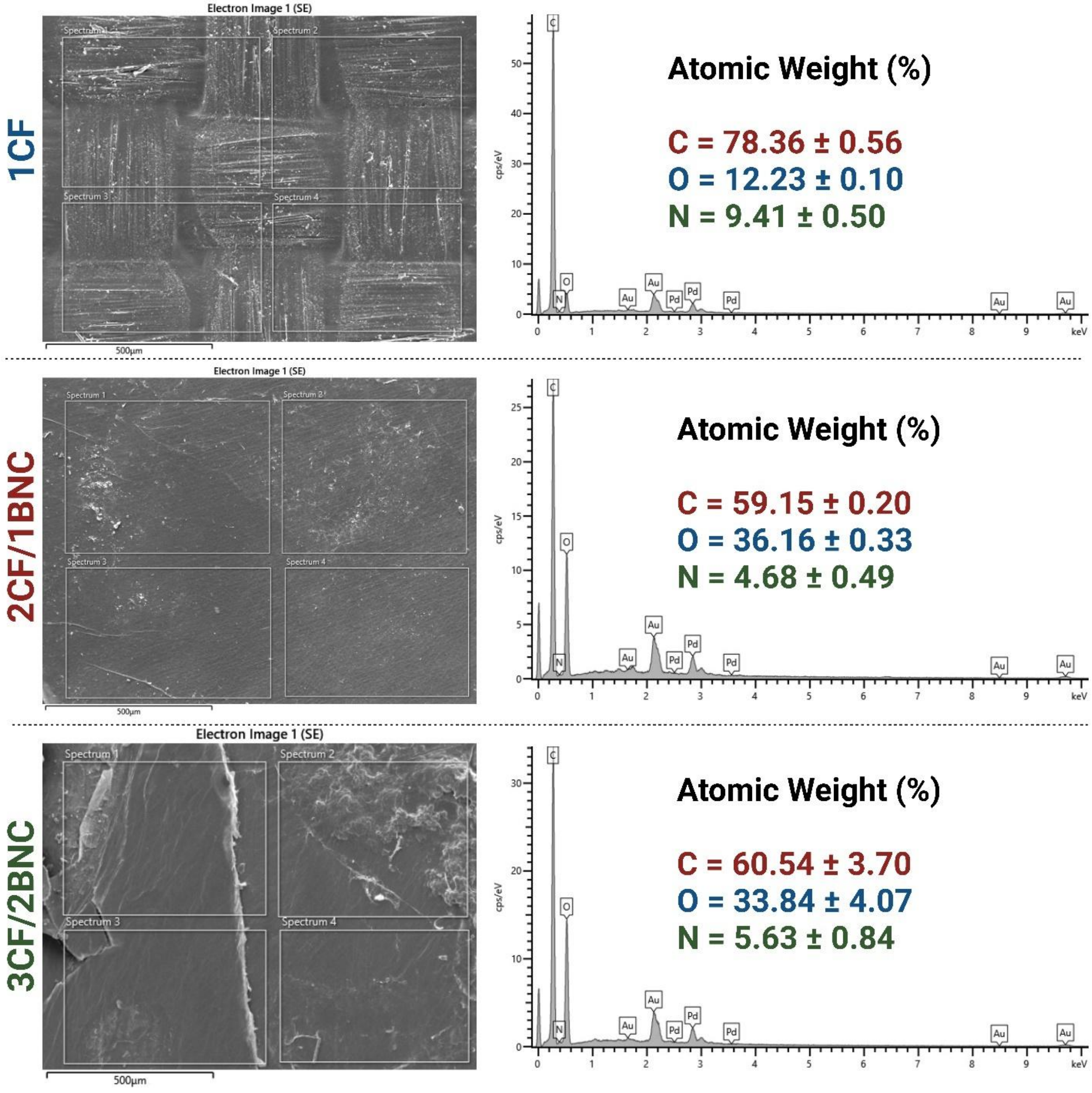

Figure S3: SEM-EDX images and elemental composition of the samples wherein 2CF/1BNC and 3CF/2BNC with exposed BNC layers.

Table S1: Samples composition and curing parameters used												
Samples	Composition		Nomenclature	Curing Parameters						Dimensions used for DMA		
	CF	BNC		Pre-Curing		On-Curing		Post-Curing		L	W	T
				Temp. (°C)	Time (h)	Temp. (°C)	Time (h)	Temp. (°C)	Time (h)	mm	mm	mm
1	1-ply	0-ply	1CF	80	1	120	4	25	1	52.28 ± 0.12	11.09 ± 0.17	0.40 ± 0.005
2	2-ply	1-ply	2CF/1BNC	80	1	120	4	25	1	52.27 ± 0.12	11.28 ± 0.08	0.50 ± 0.005
3	3-ply	2-ply	3CF/2BNC	80	1	120	4	25	1	51.73 ± 0.66	10.71 ± 0.64	0.63 ± 0.019

CF – Carbon Fiber
BNC – Bacterial Nanocellulose
L – length
W – Width
T – thickness

Table S2: Surface Topography and Roughness Measurement			
Samples	KLA Zeta Analyzer		
	Max peak height- Rp (µm)	Max Valley Depth – Rv (µm)	Max Peak to Valley Height – Rt (µm)
1CF	1.261	0.9441	2.163
2CF/1BNC	1.79	1.511	3.301
3CF/2BNC	2.061	1.574	3.634

Table S3. Comparison of Measured Contact Angle (Water) to other existing known carbon fiber reinforced polymer or composites			
Sample	Contact Angle (^{0})	Assessment	Reference
3CF/2BNC	106.46	Hydrophobic	This Study
GO@PDA@CF	88.7	Hydrophilic	[12]
Untreated CFR-PEEK	92	Hydrophobic	[13]
SGL 10BA (carbon fiber, 400 µm thick, 5 wt % PTFE, 88% porosity)	102	Hydrophobic	[14]
Oxidized PAN-carbon fiber composite	80.80	Hydrophobic	[15]
MWCNT/HA/CF/PEEK	31.6	Hydrophilic	[16]
CF-HP-COOH	42.17	Hydrophilic	[17]

Table S4: Degree of Crystallinity (%) and Crystallite Size from XRD			
Samples	Degree of Crystallinity (%)	Crystallite Size (nm)	
		(002)	*(100)*
1CF	81.86	0.91	1.42
2CF/1BNC	73.35	1.43	2.28
3CF/2BNC	79.13	1.56	2.24

Table S5: Kinetic and Thermodynamic Parameters from Thermogravimetric Analysis						
Samples	First Degradation					
	T(K)	A (min^{-1})	Ea ($kJmol^{-1}$)	ΔS ($Jmol^{-1}K^{-1}$)	ΔH ($kJmol^{-1}$)	ΔG ($kJmol^{-1}$)
1CF	652.22	3.3	9.72	-317.82	4.302	211.6
2CF/1BNC	657.05	3.36	10.04	-315.99	4.574	212.2
3CF/2BNC	649.90	5.88	17.27	-299.83	11.86	206.7
	Second Degradation					
	T(K)	A (min^{-1})	Ea ($kJmol^{-1}$)	ΔS ($Jmol^{-1}K^{-1}$)	ΔH ($kJmol^{-1}$)	ΔG ($kJmol^{-1}$)
1CF	702.86	54.18	185.4	-17.78	179.6	192.1
2CF/1BNC	708.96	48.12	167.5	-45.66	161.6	193.9
3CF/2BNC	710.32	2.64	9.16	-329.28	3.25	237.1
	Residual Yield					
	T(K)	A (min^{-1})	Ea ($kJmol^{-1}$)	ΔS ($Jmol^{-1}K^{-1}$)	ΔH ($kJmol^{-1}$)	ΔG ($kJmol^{-1}$)
1CF	868.14	6.54	34.05	-253.55	26.84	246.9
2CF/1BNC	859.99	7.32	37.59	-246.76	30.44	242.7
3CF/2BNC	868.95	8.46	44.19	-251.66	36.96	255.6

Table S6. Comparison of Measured Endset Temperature to other existing known carbon fiber reinforced polymer or composites		
Sample	T_f (°C)	Reference
CF/BNC	435.96-437.32	This Study
CF/PPA	467	18
CF/PEEK	326	19
CF/LPP	426-463	20
PEI-PEEK sized CF	532	21
Carbon fiber—PA6	450-550	22
CFRP-PI0.99	326	23

References:

1. Gorgieva, S., Trcek, J. Bacterial Cellulose: Production, Modification and Perspective in Biomedical Applications. Nanomaterials Basel, 2019, 9, 1352.

2. Warren, R., Lajeunesse, D., “Characterization of Hydrothermal Deposition of Copper Oxide Nanoleaves on Never-Dried Bacterial Cellulose,” Polymers, 2019, 11, 1762.

3. Cabo, Jr., M., More, N., Alston, J.R., Laws, E., Kulkarni, R., Mohan, R.V., LaJeunesse, D.R. “Insight on the Mechanical Properties of Facile Hydrophobic-Barrier-Patterned Bacterial Nanocellulose via Self-Bonding Mechanism,” *ACS Nanosci. Au* 2025, 5, 3, 128-136.

4. Leigh, S.J.; Bradley, R.J.; Purssell, C.P.; Billson, D.R.; Hutchins, D.A.; Hong, J. A Simple, Low-Cost Conductive Composite Material for 3D Printing of Electronic Sensors. *PloS One,* 2012, 7, e49365. https://doi.org/10.1371/journal.pone.0049365

5. Resistance, Resistivity & Sheet Resistance. Link: https://medium.com/@voltera/resistance-resistivity-sheet-resistance-a816fc19870b. Accessed: Dec. 11, 2025

6. The Voltera Team, Electronics 101: Resistance, Resistivity, and Sheet Resistance. Link: https://www.voltera.io/blog/resistance-resistivity-and-sheet-resistance. Accessed: Dec. 11, 2025.

7. Dobiášová, L., Starý, V., Glogar, P., Valvoda, V. “Analysis of carbon fibers and carbon composites by asymmetric X-ray diffraction technique,” Carbon, 1999, 37, 421-425.

8. Xiaohui Ju, Mark Bowden, Elvie E. Brown, Xiao Zhang, “An improved X-ray diffraction method for cellulose crystallinity measurement,” Carbohydrate Polymers, 2015, 476-481.

9. Naga, K., Prabhakar, M.N., Song, J.I. “Synthesis of vinyl ester resin-carrying PVDF green nanofibers for self-healing applications,” Sci Rep, 2021, 11, 908.

10. Farrukh, M. A. Butt, K. M. Chong, K. Chang, W. S. “Photoluminescence emission behavior on the reduced band gap of Fe doping in CeO2-SiO2 nanocomposite and photophysical properties,” *J. Saudi Chem. Soc.,* 2019, 23, 5, 561−575.

11. Chen, C. Bu, X. Huang, D. Huang, Y.; Huang, H. “Thermal Decomposition and Kinetics Analysis of Microwave Pyrolysis of Dunaliella salina Using Composite Additives,” *Bioenergy Res.*, 2020, 13, 4, 1205−1220.

12. Li, Y., Zhao, X., Ye, L. “Reinforcing CFRP composites by formation of tailored interfacial mechanical interlocking structure on carbon fiber surface,” *J Polym Res,* 2023, 30, 150.

13. Han, X., Yang, D., Yang, C., Spintzyk, S., Scheideler, L., Li, P., Li, D., Geis-Gerstorfer, J., Rupp, F. “Carbon Fiber Reinforced PEEK Composites Based on 3D-Printing Technology for Orthopedic and Dental Applications,” JCM, 2019, 8, 240.

14. Liu, C.P., Saha, P., Huang, Y., Shimpalee, S., Satjaritanun, P., Zenyuk, I.V. “Measurement of Contact Angles at Carbon Fiber–Water–Air Triple-Phase Boundaries Inside Gas Diffusion Layers Using X-ray Computed Tomography,” ACS Applied Materials & Interfaces, 2021, 13, 20002-20013

15. Murali, T., Murad, M.S., Bakir, M., Asmatulu, R. “PAN-based fiber-reinforced carbon-carbon composites for improved fire retardancy and thermal and electrical conductivities for harsh environments,” Journal of Composite Materials, 2024, 15, 1751-1768

16. Liu, L., Zhang, Y., Ma, S., Zhu, S., Wu, S., Wei, B., Yang, G. “Preparation and Characterization of High-Strength and High-Modulus Multi-Walled Carbon Nanotube/Hydroxyapatite/Carbon Fiber/Polyetheretherketone Composites,” Applied Sciences, 2024, 14, 1723.

17. Han, P., Yang. L., Zhang, S., Gu, Z. “Constructing a Superior Interfacial Microstructure on Carbon Fiber for High Interfacial and Mechanical Properties of Epoxy Composites,” Nanomaterials, 2022, 12, 2778.

18. Wang, Qiushi, "Mechanical Properties Of Long Carbon Fiber Reinfroced Thermoplastic (Lft) At Elevated Temperature" (2012). *All ETDs from UAB*. 3261. https://digitalcommons.library.uab.edu/etd-collection/3261

19. Why CF-PEEK Thermoplastic are Replacing CF-Epoxy Thermoset Composites Link: https://www.addcomposites.com/post/why-cf-peek-thermoplastic-are-replacing-cf-epoxy-thermoset-composites. Accessed: Dec. 11, 2025.

20. Abenojar, J., Aparicio, G.M., Butenegro, J.A., Bahrami, M., Martínez, M.A. “Decomposition Kinetics and Lifetime Estimation of Thermoplastic Composite Materials Reinforced with rCFRP,” Materials, 2024, 17, 2054.

21. Yavuz, Z., Öz, Y., Ece, R.E., Öztürk, F. “Investigation of sizing materials for carbon fiber reinforced thermoplastic composites,” Journal of Thermoplastic Composite Materials, 2025, 38, 1799-1817.

22. Giżyński, M., Romelczyk-Baishya, B. “Investigation of carbon fiber–reinforced thermoplastic polymers using thermogravimetric analysis,” Journal of Thermoplastic Composite Materials, 2025, 34, 126-140.0

23. Vaganov, G., Simonova, M., Romasheva, M., Didenko, A., Popova, E., Ivan’kova, E., Kamalov, A., Elokhovskiy, V., Vaganov, V., Filippov, A., Yudin, V. “Influence of Molecular Weight on Thermal and Mechanical Properties of Carbon-Fiber-Reinforced Plastics Based on Thermoplastic Partially Crystalline Polyimide,” Polymers, 15, 2922.
